# Neuroleptic malignant syndrome secondary to olanzapine, a presentation with severe acute kidney injury requiring hemodialysis: a case report

**DOI:** 10.1186/s13256-022-03591-5

**Published:** 2022-10-06

**Authors:** Chee Keong Chang, Alvin Oliver Payus, Malehah Mohd Noh, Meryl Grace Lansing, Doreen Sumpat, Sooth Jiun Andrea Lu, Boon Teong Yew

**Affiliations:** 1 Medicine-Based Department, Faculty of Medicine and Health Science, Universiti Malaysia Sabah, Kota Kinabalu, Sabah Malaysia; 2 Department of Medicine, Hospital Queen Elizabeth, Kota Kinabalu, Sabah Malaysia

**Keywords:** Neuroleptic malignant syndrome, Acute kidney injury, Creatine kinase

## Abstract

**Background:**

We present this case to draw attention to the importance of early diagnosis in terms of life-saving, noting that greater awareness is important among healthcare professionals. Our patient developed neuroleptic malignant syndrome (NMS) after his neuroleptic drug dosage was increased. His condition was complicated by acute kidney injury (AKI) which required hemodialysis. The uniqueness of this case is that the causative agent of NMS is an atypical antipsychotic, and atypical antipsychotics are generally considered to be safer than typical antipsychotics.

**Case presentation:**

A 31-year-old Chinese man with underlying schizophrenia presented to our hospital with aggressive behavior. He was admitted to the psychiatric hospital and started on his regular medications, with an increase in the dose of olanzapine tablet from 5 to 10 mg daily. After 5 days in the ward, the patient was noted to have high fever, restlessness, confusion, increased muscle rigidity, tachycardia and tachypnoea. Antipsychotic therapy was stopped in view of suspected NMS. The first laboratory test for serum creatine kinase (CK) showed a markedly high level of this molecule. His renal profile showed raised serum creatinine in comparison to 2 months prior when the baseline serum creatinine was within the normal range. A diagnosis of NMS with AKI was made. Although the patient was given adequate intravenous fluid hydration with close monitoring of urine output, his renal function did not show improvement but continued to show a worsening trend. In view of this, he was started on urgent hemodialysis. The patient was dependent on intermittent hemodialysis before his AKI showed complete recovery. After 2 weeks, his blood test results returned to normal. He was discharged well.

**Conclusion:**

Neuroleptic malignant syndrome is a life-threatening iatrogenic medical emergency in which high index of clinical suspicion is required for diagnosis and prompt treatment.

## Background

Neuroleptic malignant syndrome (NMS) can be potentially fatal if it is left untreated or there is a delay in diagnosis [1]. It occurs more often in those who are exposed to neuroleptic medications, with men having a higher incidence than women.

The underlying pathogenesis of NMS is still not fully understood. It has been suggested that drug-induced dopamine receptor blockade in the brain likely plays a key role in triggering the syndrome, resulting in muscle rigidity and core temperature elevation [1]. Both typical and atypical antipsychotics have been associated with NMS, but high-potency typical antipsychotics, such as haloperidol, are most commonly implicated in NMS due to their higher affinity for dopamine D2 receptors [1–3].

## Case presentation

A 31-year-old Chinese man with underlying schizophrenia presented to hospital with aggressive behavior. He had a past history of schizophrenia which was diagnosed 2 years prior, but had no other past medical or surgical history. He was admitted to the psychiatric hospital and started on his regular medications, with an increase in the dose of olanzapine tablet from 5 to 10 mg daily. After 5 days in the ward, the patient was noted to have high fever (recorded at 38.9 °C), restlessness, confusion and increased muscle rigidity. He also had tachycardia, with a heart rate 120–130 beats per minute and tachypnoea, although his blood pressure was normotensive. Antipsychotic therapy was stopped in view of suspected NMS. The patient was immediately referred to the general hospital and admitted to the acute internal medical ward for further management. Further examination revealed a fully conscious man who demonstrated generalized rigidity, including all four extremities and neck. The initial laboratory test results for serum creatine kinase (CK) showed a markedly high level (256,020 U/L). His renal profile showed serum creatinine of 766.9 umol/L (baseline serum creatinine was within the normal range 2 months prior). Arterial blood gases showed compensated metabolic acidosis. Based on these clinical findings and the laboratory results, a diagnosis of NMS with acute kidney injury (AKI) was confirmed. Although the patient was given adequate intravenous fluid hydration with close monitoring of urine output, his renal function did not show improvement but continued to show a worsening trend. He was therefore started on urgent hemodialysis and was also started on oral bromocriptine 2.5 mg three times daily and oral lorazepam 1 mg three times daily upon admission. The psychiatric team was also consulted to co-manage the patient.

**Fig. 1:**
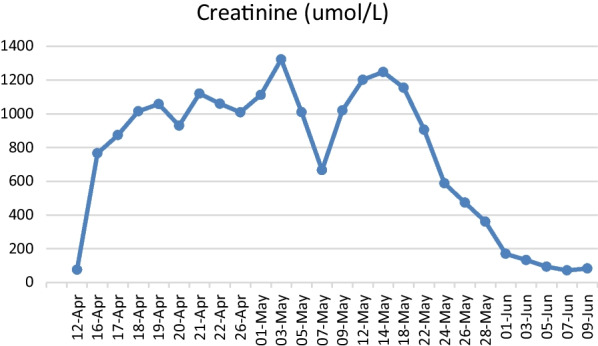
Trend in serum creatinine level. There was a sudden increase in serum creatinine. The patient was placed on intermittent hemodialysis, following which serum creatinine showed a gradual improvement back to the normal range as the acute kidney injury improved.

**Fig. 2:**
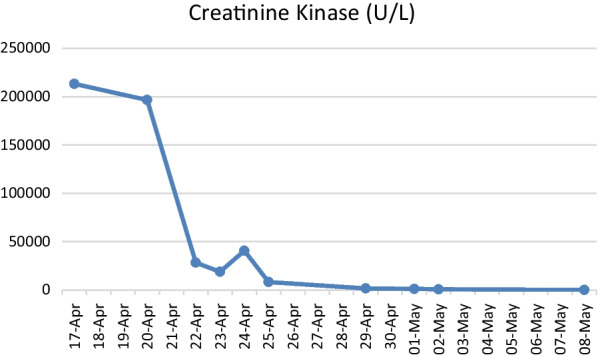
Results of the first laboratory test for creatinine kinase (CK). The CK level was extremely high when the diagnosis of neuroleptic malignant syndrome was first made, following which it showed a reducing trend

In the ward he required intermittent hemodialysis. Within a few days, his NMS symptoms improved and the CK level showed a reducing trend (Figs. 1, 2). His body temperature was back to normal after administration of the causative agent and intravenous fluid was stopped. Caution was taken, and the patient was not provided any antipsychotic drugs, including haloperidol, as these drugs might have worsened his condition. Oral lorazepam was tapered down to 0.5 mg three times daily as the patient did not show agitation. His muscle rigidity had markedly abated.

Close observation of the patient after the causative agent was stopped did not identify any psychotic symptoms. Following serial reviews by the psychiatric team in the hospital, the patient was continued on oral lorazepam at the same dose. His renal profile showed an improving trend with progressive increasing urine output. The patient was discharged well after 6 weeks of stay in the hospital. During outpatient review, it was noted that the patient had recovered from the AKI and did not require any further hemodialysis. He was kept on oral lorazepam during his outpatient review in the psychiatric clinic. He tolerated lorazepam well and did not have any symptoms, and he was able to independently carry out his daily activities and started working as a helper in a grocery shop.

A bedside ultrasound scan of the inferior vena cava was performed to help determine the fluid status of the patient before deciding on a hemodialysis regime. Close monitoring of his vital signs showed transient elevated blood pressure (highest being 155/95 mmHg) which was closely observed and did not require any antihypertensive medication.

### Timeline

The timeline of disease progression, treatment, and diagnosis of our patient is shown in Table 1.

**Table 1:** Timeline

Time	Symptom development, diagnosis, and treatments
Week 1	Received increased dose of olanzapine
	Developed symptoms of NMS, such as fever, restlessness and rigidity
	Antipsychotic medication was stopped
	Noted to have raised CK level and severe AKI. Diagnosed to have NMS
	AKI failed to respond to intravenous hydration
	Hemodialysis started
Week 2	Intermittent hemodialysis needed
Week 3	Recovery of renal function and symptoms
	Discharged well with follow-up

*AKI* Acute kidney injury,* CK* creatinine kinase,* NMS* neuroleptic malignant syndrome

## Discussion

Neuroleptic malignant syndrome is an uncommon but serious complication of neuroleptic medications. The mortality is 10–30% [4]. Approximately 16% of cases of NMS develop within 24 hours after the initiation of antipsychotic therapy, 66% within the first week, and nearly all cases within 30 days [1]. Risk factors that may precipitate NMS include substance abuse, agitation, restraints, dehydration, parenteral administration, and high doses or rapid upward titration of neuroleptics [5, 6]. In the case of the patient reported here, we noted that there was a rapid titration of olanzapine dose from 5 to 10 mg, which might be a strong factor accounting for the development of NMS. The patient also showed typical symptoms of NMS and its onset, which was within 1 week of dosage modification of medication. One possible risk factor for NMS that this patient may have been dehydrated, possibly due to a lack of awareness of the need to drink during a psychotic episode.

NMS needs to be diagnosed based on clinical suspicion by correlating clinical presentation with laboratory parameters as there is no diagnostic test for this condition [8]. A raised CK level (> 1000 U/L) is a sensitive test for NMS, but the CK level can increase to > 10,000 U/L, as in the case of our patient [9].

Once the diagnosis of NMS is suspected, the drugs possibly causing the NMS should be stopped [7–9]. Pharmalogical agents that can be used to treat muscle rigidity include dantrolene, bromocriptine, and amantadine [6]. Intravenous fluids are used for hydration and prevention of acute renal failure. An additional aim of hydration is to replace excessive fluid loss from body due to high fever. Unfortunately, our patient required hemodialysis support for his acute renal injury from myoglobinemia. Symptoms of NMS usually take 2 weeks or longer (sometimes up to 4 weeks) to resolve [8].

The pathophysiology behind the development of NMS is rhabdomyolysis. The death of muscle fibers with the subsequent release of their content, such as myoglobins, CK, and lactate dehydrogenase, will lead to the blockage of renal tubules [10]. This subsequently causes AKI. In severe cases, hemodialysis is required, as was the case of our patient. Serious complications, such as hyperkalemia resulting in cardiac arrhythmias and asystole may occur if urgent dialysis is not done.

The primary take-away lesson of this case presentation is to highlight the importance that healthcare professionals diagnose NMS as early as possible, as early detection can save lives. It is also important not to disregard atypical antipsychotic medications as one of the possible causes of NMS. In view of the fact that neuroleptic medications are commonly prescribed by psychiatrists, it essential that physicians are able to recognize NMS and make an early referral to the respective discipline for further management.

## Conclusion

Neuroleptic malignant syndrome is a life-threatening iatrogenic medical emergency in which a high index of clinical suspicion is required for diagnosis and prompt treatment.

## Data Availability

Not applicable.

## Abbreviations

AKI: Acute kidney injury
CK: Creatinine kinase
NMS: Neuroleptic malignant syndrome
